# β-Lactolin Reduces Age-Related Inflammation and Cognitive Decline

**DOI:** 10.3389/fnut.2021.724134

**Published:** 2021-08-23

**Authors:** Yasuhisa Ano, Rena Ohya, Akihiko Takashima, Kazuyuki Uchida, Hiroyuki Nakayama

**Affiliations:** ^1^Laboratory of Veterinary Pathology, Graduate School of Agricultural and Life Sciences, The University of Tokyo, Tokyo, Japan; ^2^Kirin Central Research Institute, Kirin Holdings Company Ltd., Kanagawa, Japan; ^3^Faculty of Science, Gakushuin University, Tokyo, Japan

**Keywords:** aging, cognitive decline, inflammation, β-lactoglobulin, β-lactolin, β-lactopeptide, memory, whey

## Abstract

With the rapid increase in aging populations worldwide, there has been an increase in demand for preventive and therapeutic measures for age-related cognitive decline and dementia. Epidemiological studies show that consumption of dairy products reduces the risk for cognitive decline and dementia in the elderly. We have previously demonstrated in randomized trials that the consumption of β-lactolin, a whey-derived Gly-Thr-Trp-Tyr lactotetrapeptide, improves cognitive function in older adults. Orally administered β-lactolin is delivered to the brain and inhibits monoamine oxidase, resulting in alleviation of memory impairment. However, there is currently no evidence of the effects of long-term β-lactolin intake on aging. Here, we found that the discrimination index in the novel object recognition test for object recognition memory was reduced in mice aged 20 months compared with that in young mice, indicating that age-related cognitive decline was induced in the aged mice; in aged mice fed β-lactolin for 3 months, memory impairment was subsequently alleviated. In aged mice, impairment of light/dark activity cycles was found to be induced, which was subsequently alleviated by β-lactolin consumption. Additionally, the number of activated microglia in the hippocampus and cortex and the production of cytokines (tumor necrosis factor-α, macrophage inflammatory protein-1α, and macrophage chemoattractant protein-1) were increased in aged mice compared with those in young mice but were reduced in aged mice fed β-lactolin. The age-related hippocampal atrophy was improved in aged mice fed β-lactolin. Cytochrome c levels in the hippocampus and cortex were increased in aged mice compared with those in young mice but were also reduced by β-lactolin consumption. These results suggest that β-lactolin consumption prevents neural inflammation and alleviates aging-related cognitive decline.

## Introduction

Accompanying the rapid growth of aging populations worldwide, the increasing burden of age-related cognitive impairment falls not only on patients and their families but also on national healthcare systems. In normal aging, the size of the brain decreases with age (1, 2). MRI studies have shown that gray matter volume in the prefrontal cortex and medial temporal lobe, which includes the hippocampus, declines with aging (3, 4). It is suggested that this volume loss is attributed to neuronal loss associated with aging, which changes the structure and function of synapses and neuronal networks (5). These changes in the brain are associated with age-related cognitive decline, such as memory and executive function impairment. Recent studies also show that age-related increases in inflammation are associated with cognitive decline (6). It is reported that age-related changed microglia in the aged brain shows the pro-inflammatory phenotype and reduce the protective function for neuron (7). It has been reported that the risk for cognitive decline and dementia in the elderly is associated with individual lifestyle (8); therefore, preventive approaches to cognitive decline, including dietary changes, have received increasing attention.

Recent investigations indicate that consumption of certain dairy products reduces the risk for dementia and for cognitive decline in the elderly. Ozawa et al. investigated dietary patterns and their potential association with reduced risk for dementia and cognitive decline in more than 1,000 dementia-free 60- to 79-year-old Japanese participants living in a local community (9, 10) and found that dietary patterns that included milk or dairy products reduced the risk for dementia in the general Japanese population. In addition, our previous study demonstrated that intake of Camembert cheese, a dairy product fermented by fungi, suppressed the activation of microglia and Alzheimer's disease pathologies in a 5 × FAD mouse model (11). These findings suggest that some ingredients, such as peptides, contribute to the prevention cognitive decline and dementia (12, 13).

A recent study identified β-lactolin, a β-lactoglobulin-derived Gly-Thr-Trp-Tyr tetrapeptide, which improves memory impairment in a pharmacologically-induced amnesia mouse model. β-lactolin belongs to the Trp-Tyr-related β-lactopeptide family, which also improve memory impairment and are abundant in Camembert and other types of cheeses fermented by *Penicillium* (14). Orally administered β-lactolin has been shown to be delivered to the brain, inhibit monoamine oxidase, and increase monoamine levels in the frontal cortex and hippocampus, resulting in improvements in spatial working memory and object recognition memory in mice (15–17). In addition to preclinical studies, previous clinical trials have demonstrated that supplementation with whey peptide rich in β-lactolin improves memory retrieval, attention, and executive function in the elderly (18, 19) and promotes neural activity in the cortex (20) and cerebral blood flow (21, 22). These findings suggest that consumption of β-lactolin is beneficial for the prevention of age-related cognitive decline. Furthermore, β-lactolin has been shown to suppress the activation of microglia and inflammation, which improves object recognition memory in 5 × FAD mice (23, 24). However, the effects of β-lactolin on age-related cognitive decline and age-related neuronal dysfunction have not yet been demonstrated. Most cognitive decline is associated with aging; thus, in the current study, we examined the effects of long-term consumption of β-lactolin on cognitive impairment using normally aged mice.

## Materials and Methods

### Animals

C57BL/6J male mice aged 8 and 68 weeks (Charles River Japan, Tokyo, Japan) were maintained at the Kirin Company Ltd. The Animal Experiment Committee of Kirin Company Ltd. approved all experiments, which were conducted from May 2017 to April 2019 in strict accordance with their guidelines (Approval No.; AN10607-Z00). The body weights did not differ between the aged mice groups. We made every possible effort to minimize suffering.

Mice were maintained at 23 ± 1°C under a constant 12-h light/dark cycle (light on from 8:00 a.m. to 8:00 p.m.). Behavioral pharmacological tests were performed in a sound-isolated room under the same conditions after 24 h habituation. Mice were fed a standard purified rodent diet (AIN-93M, Oriental Yeast, Tokyo, Japan) supplemented with 0, 0.05% (w/w) β-lactolin (GTWY peptide, Bachem, Bubendorf, Switzerland), or 5% (w/w) whey peptide rich in β-lactolin (whey peptide contains 1.6 mg of β-lactolin per gram and other components were amino acids and di, tripeptides Kirin Holdings Co. Ltd.), which was used in our previous clinical trials, for 3 months. Previously we showed that β-lactolin contributed to improve cognitive function as a responsible agent. Evaluation of the condition of skin and hair (glossiness, coarseness, hair loss, and ulcer) was performed using the senescence grading score system previously reported (25, 26). After each behavioral evaluation, samples taken from the brains of the mice were used for subsequent biochemical evaluations. Mice were euthanized in a chamber filled with 5% isoflurane vapor (Wako, Osaka, Japan). We tested 15 young and 15 aged mice with AIN-93M, 15 aged mice with AIN-93M containing 0.05% (w/w) β-lactolin, and 15 aged mice with 5% (w/w) whey peptide.

### Novel Object Recognition Test

To evaluate episodic memory, we performed the NORT in accordance with the methods described in our previous study (14). The NORT was performed during the light period in a polyvinyl chloride box (25 × 40 × 20 cm) without a roof. For the acquisition trial, we used a pair of wooden triangle poles (4.5 × 4.5 × 4.5 cm) or wooden pyramids (4.5 × 4.5 × 4.5 cm); for the retention trial, we used a pair of poles or pyramids and a golf ball (4.5 cm diameter). In all trials, we placed the objects 7.5 cm from the corner of the box. In the acquisition trial, mice were allowed to explore the box with the two objects for 10 min at 1 h after oral administration of the test sample. Twenty-four hours after the acquisition trial and 1 h after oral administration, the mice were permitted to explore the box with the novel and familiar objects for 5 min. The discrimination index (DI) was calculated using the following equation:

$$\begin{array}{l} \frac{novel\;object\;exploration\;time\;-\;familiar\;object\;exploration\;time}{total\;exploration\;time} \end{array}$$

Thus, equal exploration of both objects was indicated by a DI of 0.

### Measurements of Activity, Food Intake, and Water Intake in Home Cages

We monitored the amount of food intake, water intake, and ambulatory activity in the home cages using a three-point meter (O'Hara & Co., Ltd., Tokyo, Japan) for 72 h according to a previously described method (27). Activity was monitored by infrared beams positioned on the X and Y axes around the home cages; the beams automatically measured the positions of the mice and their movement. In this experiment, we measured the moving distance of each mouse every 10 min for a total of 72 h.

### Quantification of Cytokines and Chemokines

To measure cytokine levels, we homogenized the hippocampus and frontal cortex from the left hemisphere in Tris-buffered saline (Wako) with a multi-bead shocker (Yasui Kikai, Osaka, Japan). After centrifugation at 50,000 × *g* for 20 min at 4°C, we collected the supernatant. We measured the total protein concentration of each supernatant using a BCA protein assay kit (ThermoScientific, Yokohama, Japan). We assayed the supernatant to quantify cytokines and chemokines using a Bio-Plex assay system (Bio-Rad, Hercules, CA, USA).

### Immunohistochemistry

The right-brain hemispheres were fixed in 10% formalin solution (Wako), paraffin-embedded, and cut into 5 μm serial sections to evaluate infiltration of activated microglia, astrocytes, and cytochrome C. The studied brain regions included the hippocampus and cerebral cortex (bregma 2.30 mm posterior). After the sections were dewaxed and rehydrated, those for ionized calcium-binding adaptor molecule 1 (Iba-1), glial fibrillary acidic protein (GFAP), and cytochrome C measurements were autoclaved at 121°C for 10 min in 0.2% citrate buffer (pH 6.0) for antigen retrieval. The sections were then incubated with blocking solution (8% w/v skimmed milk) for 30 min after inactivation of endogenous peroxidase with 3% H_2_O_2_ (Wako) in methanol for 5 min. Subsequently, the sections were incubated overnight at room temperature with primary antibodies: polyclonal anti-Iba-1 antibodies (Wako, 1: 500), polyclonal anti-GFAP antibodies (Dako, Glostrup, Denmark, 1: 500), or monoclonal anti-cytochrome C antibodies (Santa Cruz, CA, USA, 1: 100). After the sections were incubated with horseradish peroxidase-coupled goat anti-mouse or rabbit IgG antibodies (4 μg/mL, Nichirei, Tokyo, Japan) for 1 h at room temperature, they were visualized with 3,3'-diaminobenzidine (Wako) and counterstained with hematoxylin. The size of the positive region per area evaluated was measured using Image J image analysis software (National Institutes of Health, Bethesda, MD, USA).

### Statistical Analyses

Data are presented as the mean and the error bars as the standard error of the mean. We analyzed data by one-way analysis of variance, followed by the Tukey–Kramer test or analyzed data by student's *t-*test. All statistical analyses were performed with the Ekuseru-Toukei 2012 software program (Social Survey Research Information, Tokyo, Japan). We considered a *p*-value < 0.05 to be statistically significant.

## Results

### Preventive Effects of β-Lactolin on Age-Related Memory Impairment in Aged Mice

**Figure 1:**
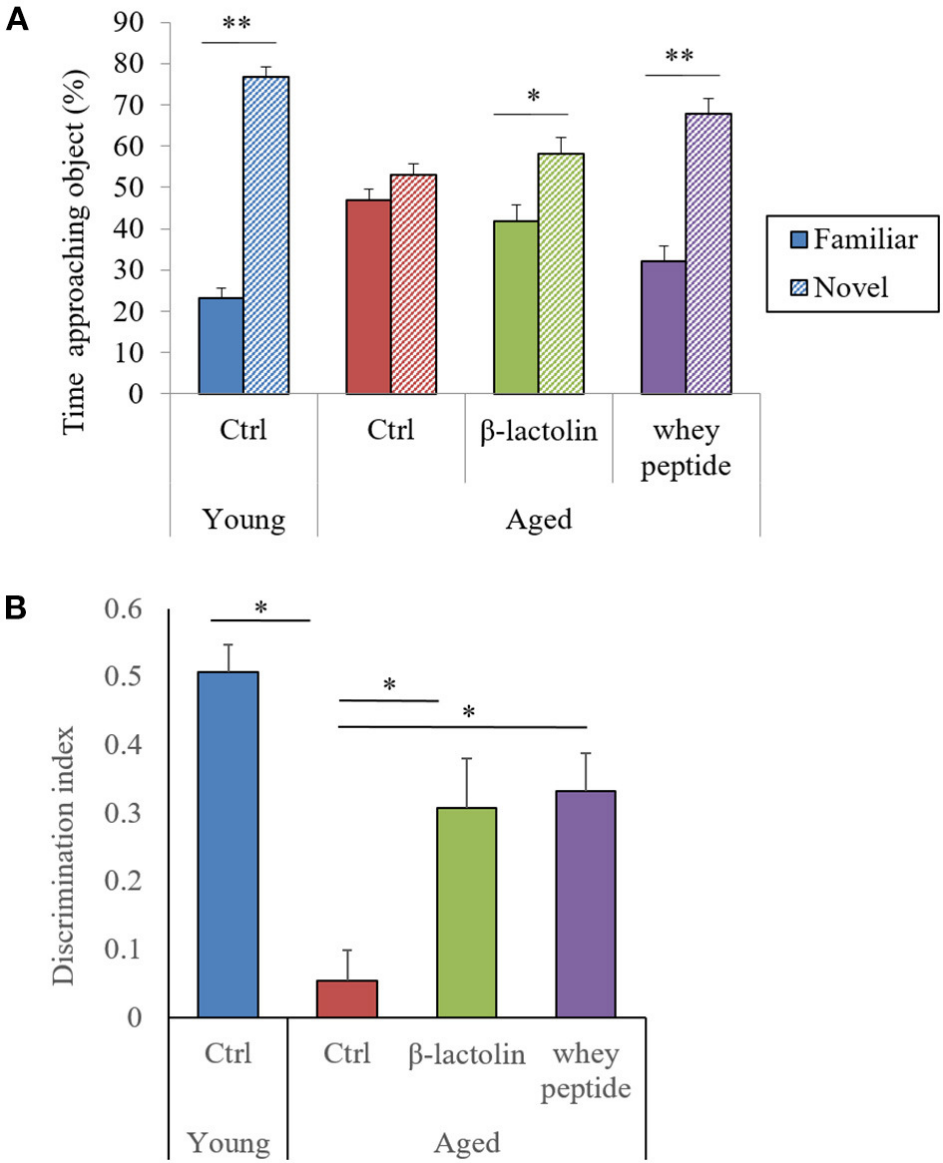
Novel object recognition test. The time taken to approach a novel or familiar object was measured **(A)**, and the discrimination index **(B)** in the novel object recognition test was calculated. Data are represented as mean ± SE and were analyzed by Student *t*-test **(A)** and one-way ANOVA, followed by the Tukey–Kramer test **(B)**. ^*^p < 0.05 and ^**^p < 0.01.

To evaluate the effects of β-lactolin and whey peptide rich in β-lactolin on memory impairment in aged mice, we fed mice aged 8 weeks (young) and 65 weeks (aged) a diet containing β-lactolin or whey peptide for 3 months and subjected them to behavioral memory evaluations using the NORT. The time taken by young mice to approach a novel object was significantly longer than that to approach a familiar object, but this difference was not observed at a significant level in the control aged mice; however, it was observed in the aged mice fed β-lactolin or whey peptide (Figure 1A). This difference in approaching time between familiar object and novel object was greater in aged mice with whey peptide than those with β-lactolin. There was not significantly different in approaching time for novel object among the groups. The DI for control aged mice was significantly decreased compared with that for young mice, and for aged mice fed with β-lactolin or whey peptide, the DIs were significantly increased compared with that for control aged mice (Figure 1B). The total time taken to approach each object did not differ among groups. These results indicate that object recognition memory in mice 80 weeks old had declined with age.

### Effects of β-Lactolin on Locomotor Activity During Light/Dark Cycles in Aged Mice

**Figure 2:**
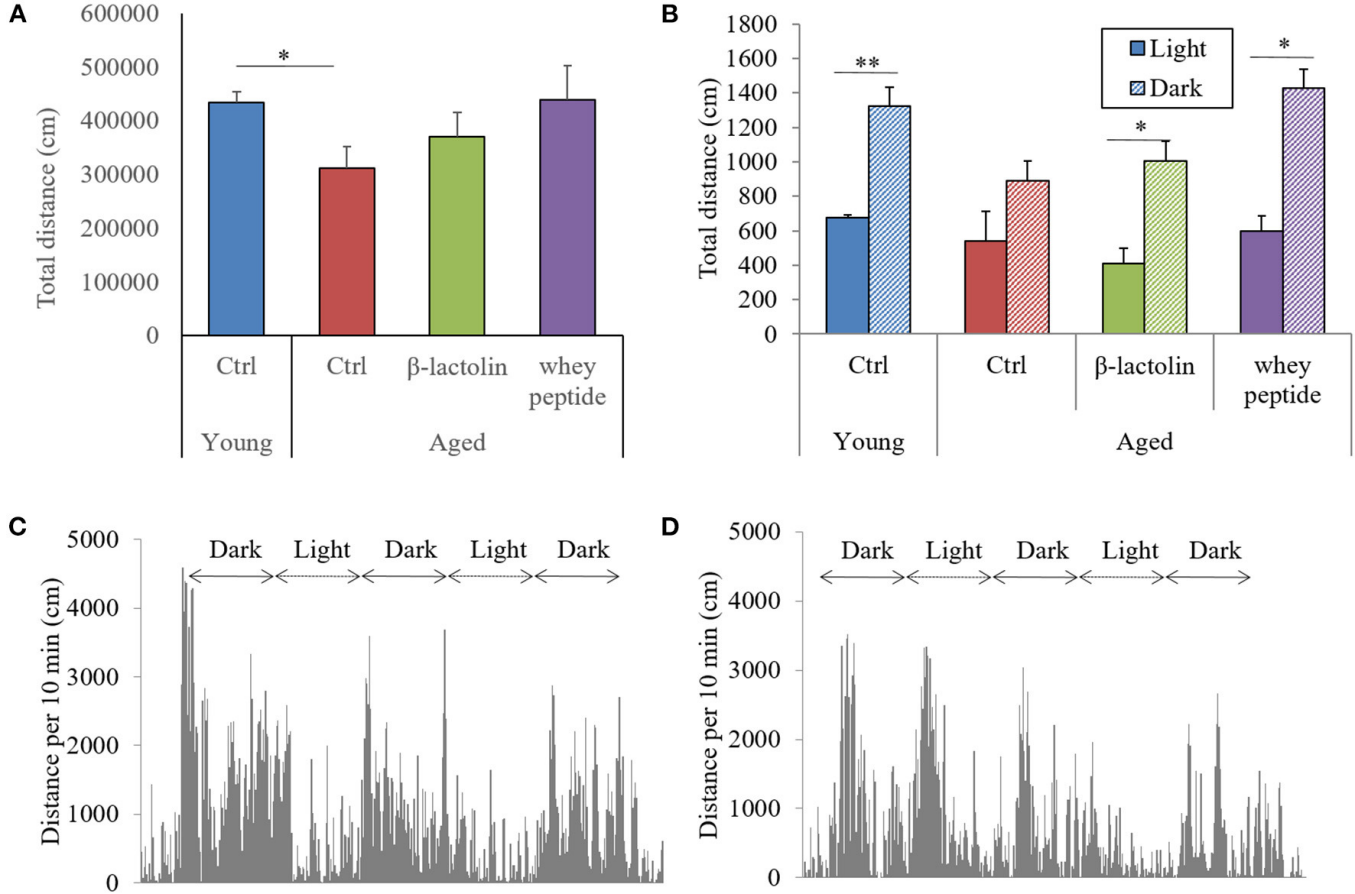
Measurement of activity during light/dark cycles. Distance traveled every 10 min over 72 h. Total distance in the home cage over 72 h **(A)** and the distance over 10 min during light and dark periods **(B)**. Distance grap every 10 min over 72 h. by young mice **(C)** and aged control mice **(D)**. Data are represented as mean ± SE and were analyzed by Student *t*-test **(B)** and one-way ANOVA, followed by the Tukey–Kramer test **(A)**. ^*^p < 0.05 and ^**^p < 0.01.

To evaluate the effects of β-lactolin and whey peptide rich in β-lactolin on locomotor activity in aged mice, we monitored the activity of mice in their home cages for 72 h. The total distance traveled over 72 h by control aged mice was significantly decreased compared with that traveled by young mice, and this reduction was attenuated by β-lactolin and whey peptide (Figure 2A). Young mice showed a distinctive cycle of locomotor activity, which was significantly higher during dark periods than during light periods. This cycle was not observed in control aged mice (Figure 2B). Young mice showed a distinctive cycle of locomotor activity per 10-min period, which was higher during dark periods and lower during light periods (Figure 2C); the amplitude of this cycle was, however, diminished in aged mice (Figure 2D). On the other hand, this light-dark cycle was observed in aged mice fed β-lactolin or whey peptide (Figure 2B). However, there was no significant difference on total distance during light or dark periods among the group. These results indicate that activity and sleep cycles were impaired in aged mice; compared with control aged mice, aged mice fed β-lactolin or whey peptide showed improvements in these impairments. The amounts of food and water intake did not differ significantly among the groups (Supplementary Figures 1A,B). The body weight among the aged groups was not changed (Supplementary Figure 1C). Notably, the weight of the hippocampus was significantly reduced in aged mice compared with that in young mice, and in aged mice fed β-lactolin or whey peptide, it was increased compared with that in control aged mice (Supplementary Figure 1D).

### Effects of β-Lactolin on Glial Activation and Cytochrome C in Aged Mice

**Figure 3:**
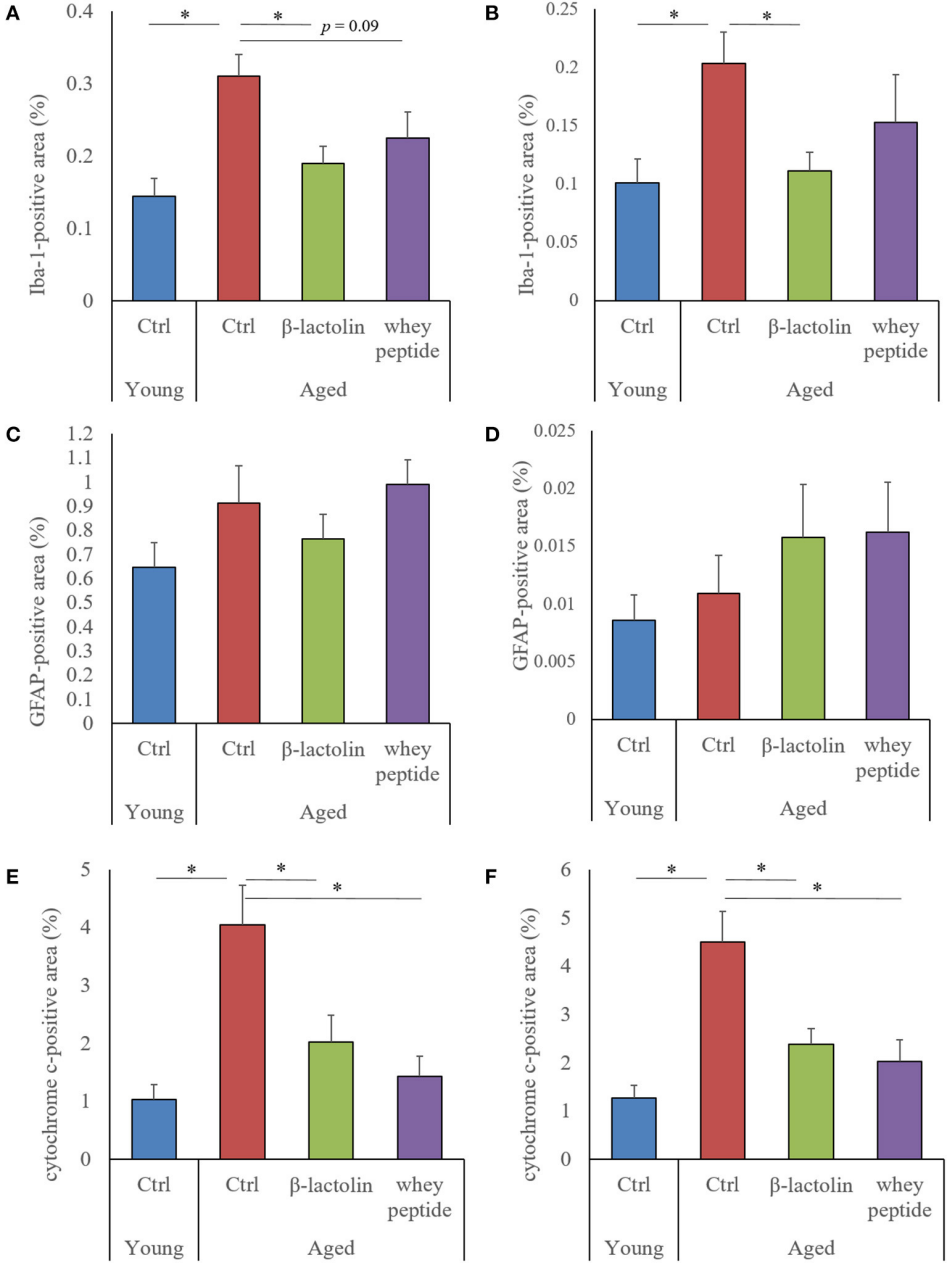
Measurement of glial activation and cytochrome C in aged mice. In the hippocampus and cerebral cortex: percentage of the Iba-1-positive area (**A,B**, respectively); percentage of the GFAP-positive area (**C,D**, respectively); percentage of the cytochrome C-positive area (**E,F**, respectively). Areas were detected by immunohistochemistry. Data are represented as mean ± SE and were analyzed by one-way ANOVA, followed by the Tukey–Kramer test. ^*^p < 0.05.

To evaluate the age-induced effects of β-lactolin and whey peptide rich in β-lactolin on glial activation and cytochrome C, we performed immunohistochemical analysis. The positive areas of Iba-1 in the hippocampus and cerebral cortex of control aged mice were significantly increased compared with those in young mice (Figures 3A,B, respectively). The Iba-1 positive areas in the hippocampus and cerebral cortex of aged mice fed β-lactolin were significantly lower than those in control aged mice (Figures 3A,B, respectively), and in the hippocampus of aged mice fed whey peptide, it tended to be significantly lower than that in control aged mice. The representative images in young mice, control aged mice, and aged mice fed with β-lactolin or whey peptide were shown in Supplementary Figures 2A–D, respectively. The positive areas of GFAP in the hippocampus and cerebral cortex did not show significant differences between young mice and aged mice (Figures 3C,D, respectively). The positive areas of cytochrome C in the hippocampus and cerebral cortex of the control aged mice were significantly increased compared with those of young mice and were significantly reduced in aged mice fed β-lactolin or whey peptide (Figures 3E,F, respectively).

### Effects of β-Lactolin on Cytokine and Chemokine Production in Aged Mice

**Figure 4:**
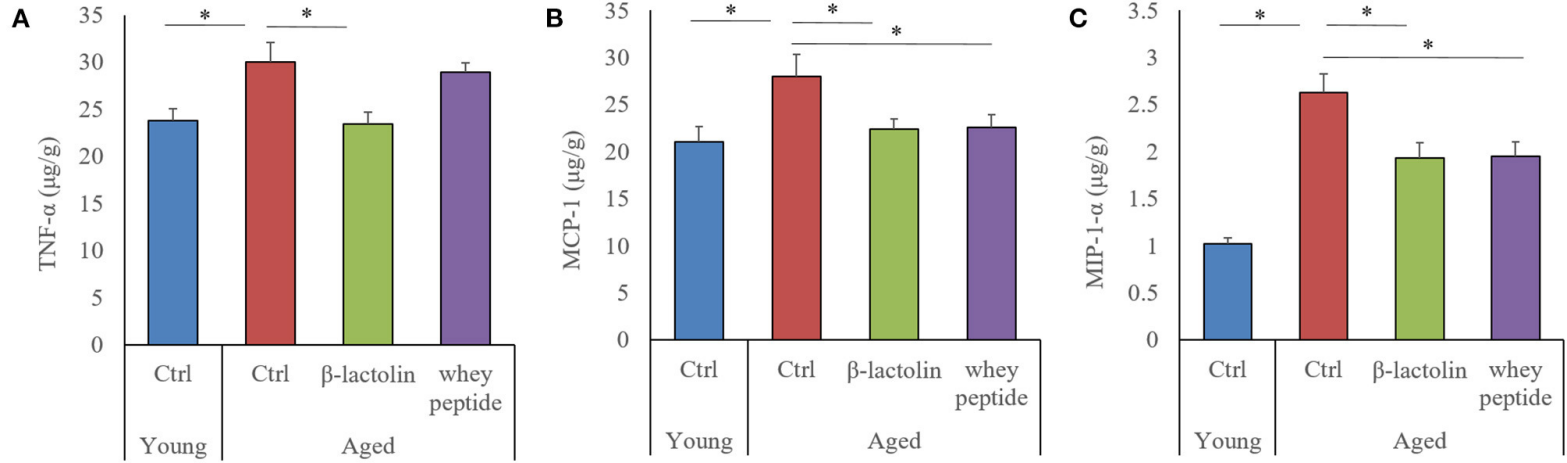
Levels of cytokines and chemokines in the hippocampus of aged mice. The levels of TNF-α, MCP-1, and MIP-1α in the hippocampus (**A–C**, respectively). Data are represented as mean ± SE and were analyzed by one-way ANOVA, followed by the Tukey–Kramer test. ^*^p < 0.05.

The levels of tumor necrosis factor-α (TNF-α), macrophage chemoattractant protein-1 (MCP-1), and macrophage inflammatory protein-1α (MIP-1α) were measured to evaluate the age-induced effects of β-lactolin and whey peptide rich in β-lactolin on these proteins in the hippocampus of aged mice. The levels of TNF-α, MCP-1, and MIP-1α in the hippocampus of aged mice were significantly increased compared with those in the hippocampus of young mice (Figures 4A–C, respectively). The level of TNF-α in aged mice fed β-lactolin and the levels of MCP-1 and MIP-1α in aged mice fed β-lactolin or whey peptide were significantly reduced compared with those in control aged mice. These results indicate that age-related inflammation was induced in the hippocampus of aged mice, and those reactions were reduced by consumption of β-lactolin or whey peptide rich in β-lactolin.

## Discussion

Our study demonstrated that the consumption of β-lactolin or whey peptide rich in β-lactolin reduced age-related inflammation in the brain and improved memory impairment associated with aging. Aged mice displayed microglial activation, increases in levels of inflammatory molecules and apoptotic molecules of cytochrome C in the hippocampus and cerebral cortex, impairment of object recognition memory, and disruption of light/dark activity circadian cycles; these effects were improved by the administration of β-lactolin.

In aged mice, microglial inflammation has been reported to be induced by Aβ and/or other antigens; activated microglia produce pro-inflammatory cytokines and reactive oxygen species, both of which lead to cognitive impairment (28, 29). Our previous study showed that the levels of Aβ and glutamate in the hippocampus in aged mice were increased compared with young mice (27). Moreover, object recognition memory, assessed by the NORT in the present study, is a hippocampus-dependent type of memory (30–32). Inflammation in the hippocampus is reported to impair object recognition memory, and the suppression of inflammation subsequently improves these impairments (33, 34). These reports therefore suggest that β-lactolin and whey peptide rich in β-lactolin suppressed microglial inflammatory responses, including the production of the pro-inflammatory cytokine TNF-α and the chemokines MCP-1 and MIP-1α, resulting in the prevention of hippocampal cognitive decline associated with aging. In the current study, the preventive effects of whey peptide on microglia infiltration and TNF-α production was not observed, which was observed in the β-lactolin group. Recent study showed that BCAA (branched-chain amino acids) rich in whey peptide increase reactive oxygen and inflammation (35), which might affect the differences on the results between β-lactolin group and whey peptide group.

The current study also showed that cytochrome C is increased in the hippocampus and cerebral cortex and that the weight of the hippocampus was reduced in aged mice compared with these parameters in young mice. Cytochrome C is known as a mitochondria-related molecule that activates caspase signaling to induce apoptosis and is associated with inflammation (36). Mitochondrial dysfunction has been reported to be associated with hippocampal atrophy and memory impairment (37). Consequently, it is suggested that β-lactolin and whey peptide rich in β-lactolin may improve mitochondrial dysfunction, subsequently improving inflammatory responses and hippocampal atrophy and resulting in the improvement of cognitive function.

In our study, we further demonstrated the impairment of light/dark activity cycles in aged mice. Circadian rhythms are reportedly impaired in aged mice via sympathetic dysfunction in peripheral clock regulation (38). Neuroinflammation has also been associated with the dysfunction of circadian rhythms (39). The regulation of inflammation in the brain by β-lactolin and whey peptide rich in β-lactolin might improve age-related impairment of light/dark activity cycles. The current study also showed that β-lactolin and whey peptide rich in β-lactolin suppressed the increases in senescence scores in aged mice (Supplementary Figure 3). β-lactolin may help prevent not only brain inflammation and cognitive decline but also other senescence phenotypes. Further study using aged mice is required to investigate these effects in detail.

Epidemiological studies have shown that regular intake of dairy products prevents cognitive decline in the elderly (9, 10), andour previous randomized clinical trials demonstrated that, compared with placebo, supplementation with whey peptide rich in β-lactolin for 6 or 12 weeks improves memory impairment (18, 19) and enhances cerebral blood flow (21, 22) and neural activity (20) in elderly with subjective cognitive decline. These reports expect that supplementations with β-lactolin for a long period is beneficial to prevent the age-related cognitive decline, on the other hand, the effects of long-term supplementation with β-lactolin, which is abundant in fermented dairy products, on age-related cognitive decline have not been investigated. As a mechanisms, in a pharmacokinetics study using radioisotope-labeled β-lactolin, orally administered β-lactolin was smoothly absorbed into the blood and delivered to each brain region (14). A previous study showed that β-lactolin inhibits the activity of monoamine oxidase B and increases the level of dopamine (14), neither of which is directly involved in inflammation. However, β-lactolin might be also associated with other molecular inflammation-related targets, such as mitochondria. High levels of some cytokine have been suggested to be associated with dementia and cognitive decline (40). The current study speculates that long-term supplementation with β-lactolin will be beneficial for the prevention of cognitive decline. Further clinical trials should investigate the effect of β-lactolin on inflammatory molecules and mitochondria-related molecules in the blood to elucidate the mechanism in humans.

The current study has some limitations. We performed novel object recognition test and Y-maze test to evaluate the memory function, whereas we could not detect the age-related deficit in Y-maze test. Further study need to evaluate another behavioral evaluation such as Morris water maze. We observed the difference of the activities during light and dark cycle between the young mice and aged mice but did not evaluate the circadian gene expressions including PERs and CRYs. To discuss the effects of β-lactolin on aged-related circadian rhythms, further study need to measure the gene expressions. We also observed β-lactolin suppressed the cytochrome c in aged mice, suggesting that β-lactolin improves the mitochondria related apoptosis, but did not evaluate the effects of β-lactolin on the activity of mitochondria. Further study need to evaluate the effects of β-lactolin on mitochondria.

In conclusion, our study demonstrated that β-lactolin, a β-lactoglobulin-derived lactopeptide, prevents age-related inflammation in the brain and cognitive decline in aged mice. The results of the current study support those of previous epidemiological studies as well as our previous reports. The consumption of β-lactolin and whey peptide rich in β-lactolin is a safe and easy practice to adopt in daily life and may be a beneficial approach to the prevention of aging-related brain disorders.

## Data Availability Statement

The raw data supporting the conclusions of this article will be made available by the authors, without undue reservation.

## Ethics Statement

The animal study was reviewed and approved by The Animal Experiment Committee of Kirin Company Ltd.

## Author Contributions

YA conducted the experiment and wrote the manuscript. RO did the experiment in the behavioral evaluations. AT, KU, and HN designed and conducted this research. All authors contributed to the article and approved the submitted version.

## Conflict of Interest

YA and RO were employed by company Kirin Holdings Company Ltd. This study received funding from Kirin Holdings Company Ltd. The funder had the following involvement with the study: the decision to publish. The remaining authors declare that the research was conducted in the absence of any commercial or financial relationships that could be construed as a potential conflict of interest.

## Publisher's Note

All claims expressed in this article are solely those of the authors and do not necessarily represent those of their affiliated organizations, or those of the publisher, the editors and the reviewers. Any product that may be evaluated in this article, or claim that may be made by its manufacturer, is not guaranteed or endorsed by the publisher.

## Supplementary Material

Supplementary Figure 1 Food and water consumption and body weight in aged mice. Food **(A)** and water **(B)** consumption over 72 h was monitored. Body weight and hippocampal weight **(C,D)**. Data are represented as mean ± SE and were analyzed by two-way ANOVA, followed by the Tukey–Kramer test.

Supplementary Figure 2 Measurement of glial activation in aged mice. Iba-1-positive microglia and GFAP-positive astrocyte were detected by immunohistochemistry. **(A–D)** Representative immunohistochemistry images for Iba-1 in youg mice, control aged mice, and aged mice fed β-lactolin or whey peptide, respectively. **(E–H)** Representative immunohistochemistry images for GFAP in youg mice, control aged mice, and aged mice fed β-lactolin or whey peptide, respectively. Scale bars, 400 μm.

Supplementary Figure 3 Senescence scores for aged mice. Senescence scores in the skin and hair category are shown. Data are represented as mean ± SE and were analyzed by one-way ANOVA, followed by the Tukey–Kramer test. ^*^*p* < 0.05 and ^**^*p* < 0.01.

## Abbreviations

NORT: novel object recognition test
DI: discrimination index
GFAP: glial fibrillary acidic protein.
