# Supplementary figures and images for: β-Lactolin Reduces Age-Related Inflammation and Cognitive Decline
Source: Front Nutr. 2021 Aug 23;8:724134. doi: 10.3389/fnut.2021.724134 (PMC8419277; doi:10.3389/fnut.2021.724134)

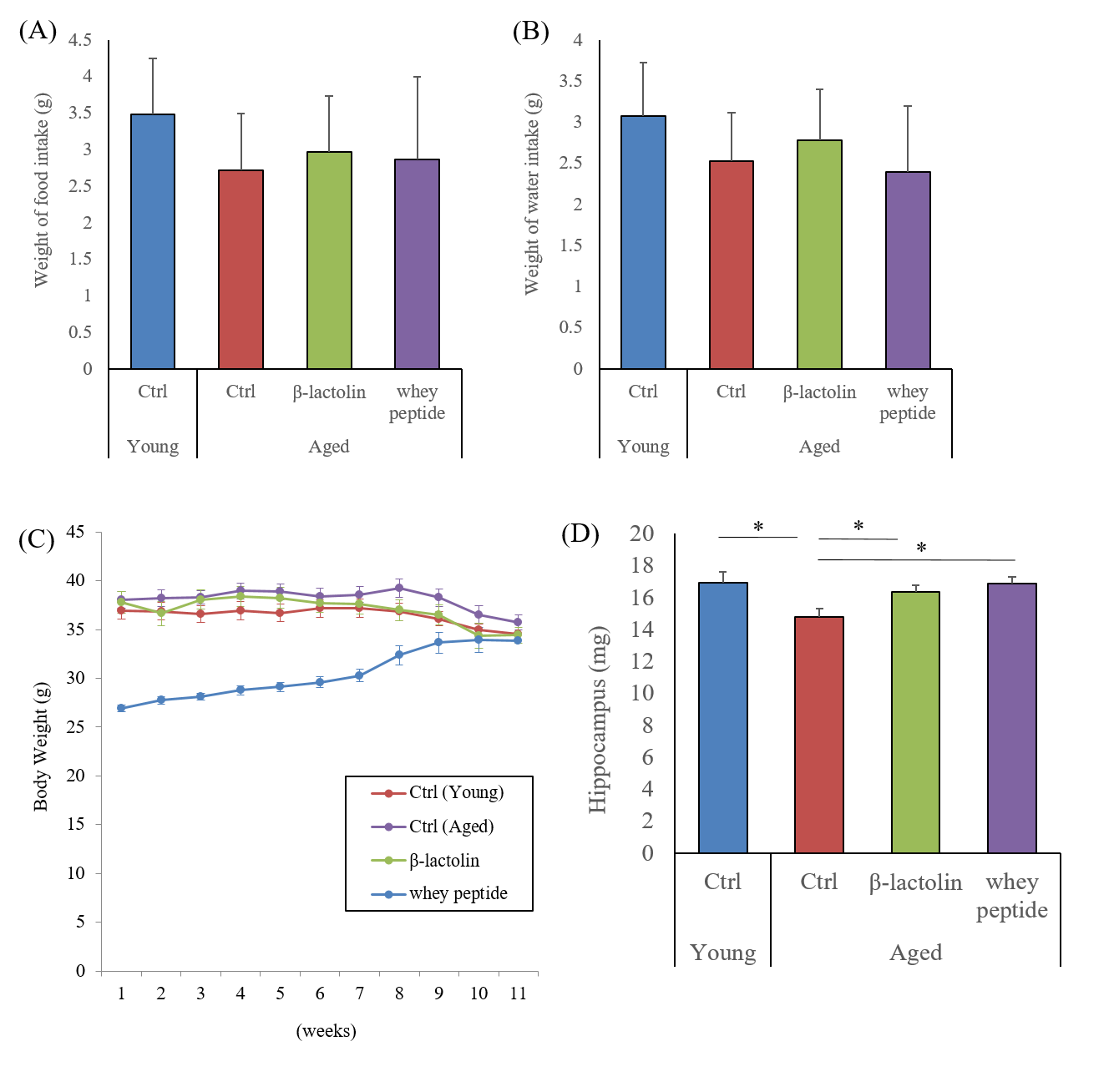

Supplement: Supplementary Figure 1 — Food and water consumption and body weight in aged mice. Food (A) and water (B) consumption over 72 h was monitored. Body weight and hippocampal weight (C,D). Data are represented as mean ± SE and were analyzed by two-way ANOVA, followed by the Tukey–Kramer test. [file Image_1.TIF]

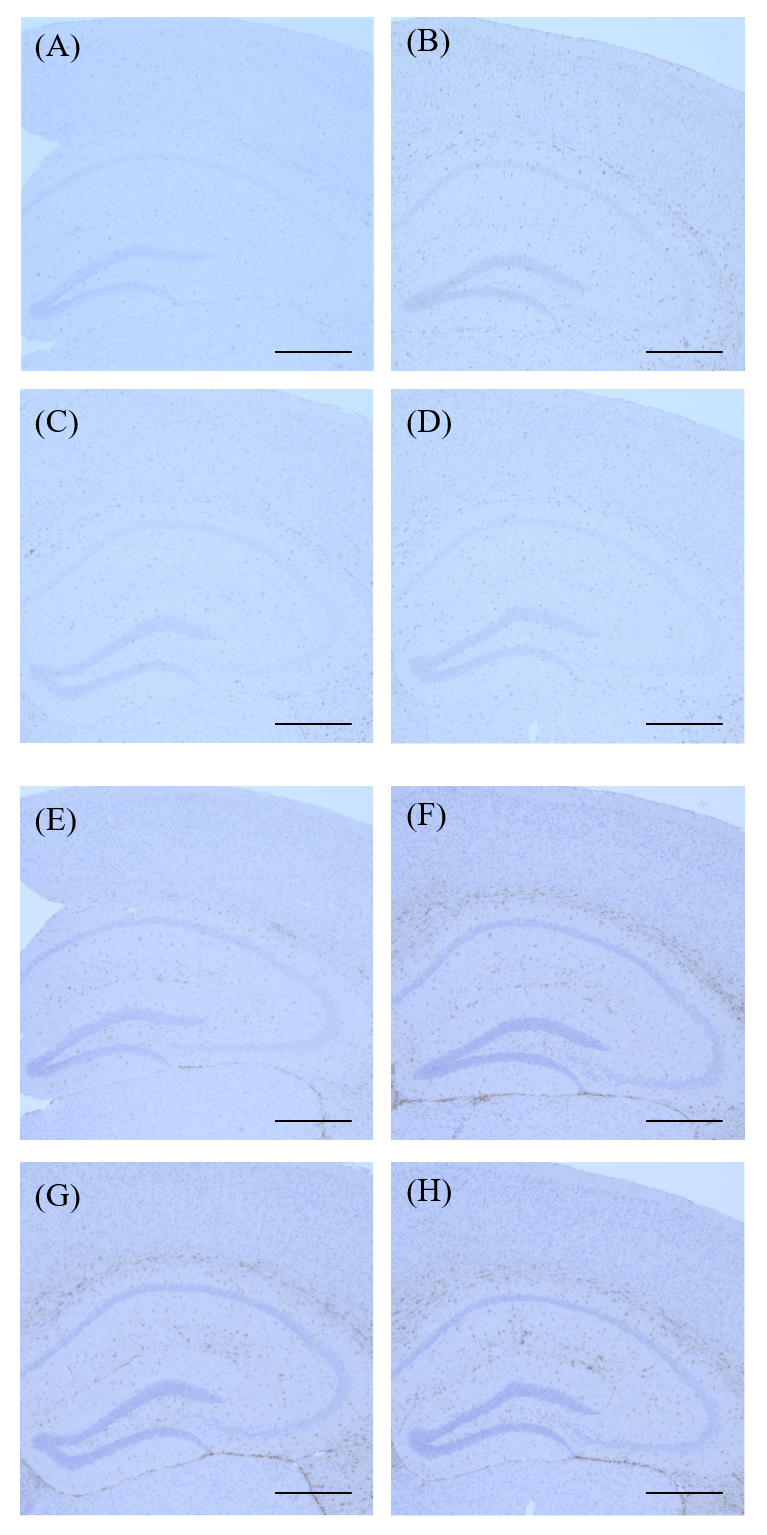

Supplement: Supplementary Figure 2 — Measurement of glial activation in aged mice. Iba-1-positive microglia and GFAP-positive astrocyte were detected by immunohistochemistry. (A–D) Representative immunohistochemistry images for Iba-1 in youg mice, control aged mice, and aged mice fed β-lactolin or whey peptide, respectively. (E–H) Representative immunohistochemistry images for GFAP in youg mice, control aged mice, and aged mice fed β-lactolin or whey peptide, respectively. Scale bars, 400 μm. [file Image_2.TIF]

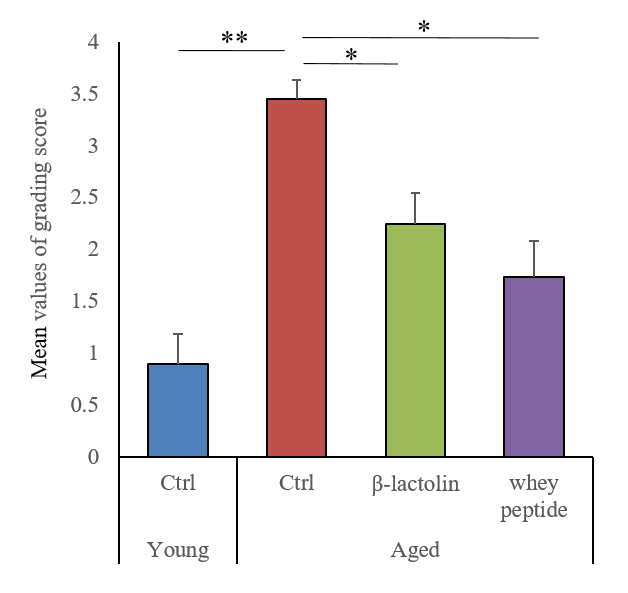

Supplement: Supplementary Figure 3 — Senescence scores for aged mice. Senescence scores in the skin and hair category are shown. Data are represented as mean ± SE and were analyzed by one-way ANOVA, followed by the Tukey–Kramer test. *p < 0.05 and **p < 0.01. [file Image_3.TIF]
